# Clinical features of *Mycoplasma pneumoniae* pneumonia in children without fever

**DOI:** 10.1186/s12887-023-04512-1

**Published:** 2024-01-16

**Authors:** Jialin Li, Hua Zhang, Jing Guo, Xiang Ma

**Affiliations:** 1 Department of Respiratory Disease, Children’s Hospital affiliated to Shandong University, Jinan, 250022 Shandong China; 2 Jinan Key Laboratory of Pediatric Respiratory diseases, Jinan Children’s Hospital, Jinan, China; 3 Shandong Provincial Clinical Research Center for Children’s Health and Disease, Jinan, 250022 Shandong China

**Keywords:** Fever, *Mycoplasma pneumoniae*, Pediatric, Respiratory function tests, Signs and symptoms

## Abstract

**Background:**

*Mycoplasma pneumoniae* (MP) is one of the most common causes of community-acquired pneumonia in children. Most children have fever. In 2021, we found that the proportion of children without fever increased. The aim of this study is to summarize the differences in the clinical characteristics of children with MP pneumonia who are febrile or not, and to raise awareness of children who are not febrile.

**Method:**

Demographic information of the children was collected on admission. Clinical manifestations during the course of the disease and the first laboratory, imaging, and pulmonary function tests before discharge were recorded and compared.

**Results:**

From August to December, a total of 542 people were included in the study. We found that older children were more likely to have fever. Inflammatory indicators including procalcitonin (*P* = 0.030), C-reaction protein (*P* < 0.001), erythrocyte sedimentation rate (*P* < 0.001), ferritin (*P* = 0.040) and the rate of atelectasis (*P* = 0.049) of febrile children were higher in febrile children. However, the elevated lactate dehydrogenase and pulmonary function impairment (*P* all > 0.05), especially the small airway function impairment, are no lower in afebrile children than in febrile children.

**Conclusion:**

The fever rate is lower in younger children, but wheezing is more common. In afebrile children, the impairment of organ and lung function was no less than in febrile children. Therefore, attention should also be paid to children who are not febrile.

## Background

*Mycoplasma pneumoniae* pneumonia (MPP) is one of the most common community-acquired pneumonia (CAP) in children, accounting for 10–40% of CAP cases in hospitalized children [1–3]. MPP epidemics are reported to occur cyclically in 3–7 year intervals [4, 5]. It presents with a variety of manifestations, such as fever, cough, wheezing and vomiting [6]. Although sometimes considered a self-limiting disease, MPP can lead to hospitalization, impaired pulmonary function damage and even some serious complications [7]. It has been reported that most children will have fever during the course of the disease [8]. In the winter of 2021, we noticed a significant increase in the incidence of MPP in all children with pneumonia. Some of them did not have obvious fever but still had significant clinical symptoms, inflammatory responses and persistent pulmonary function impairment. However, it’s unclear whether there is a difference in the clinical presentation of children with MPP who are febrile or not. Therefore, in this retrospective study, we compared the general information, clinical manifestations, laboratory and imaging examinations, and pulmonary function tests between febrile and afebrile children to draw the attention of pediatricians to afebrile children with MPP.

## Method

### Study design and population

We counted the children with MPP who were admitted to the respiratory department of our hospital in 2021. Then, we performed a retrospective analysis of a total of 542 children from August to December 2021. This period was the peak incidence of MPP in that year. As this is a retrospective observational study, all people with a diagnosis of MPP were included. In the present study, the diagnosis of MPP was based on *Zhu Futang Practice of Pediatrics* (8th Edition) [9] and the expert consensus on MPP (2015) [10], and the criteria included the following: (1) fever, cough and other respiratory tract infection symptoms and/or other extrapulmonary manifestations; (2) moderate and fine moist rales heard in the lungs by auscultation and/or lung lesions found by imaging; and (3) MP-IgM positive by enzyme-linked immunosorbent assay (ELISA) or MP-IgM or IgG antibody titer increased or decreased by 4-fold or more in the convalescent and acute phases or changed from negative to positive by fluorescence quantitative polymerase chain reaction (PCR).

All patients were divided into a febrile group and an afebrile group according to whether they had fever throughout the whole course of disease (including before and after admission). The standard for assessing fever is the uniform use of a mercury thermometer to measure axillary temperature, and below 37.3℃ is the normal temperature.

### Demographic and clinical data

Demographic data of all the patients were analyzed. The presence of wheezing and atelectasis during the course of the disease was recorded. The examination data of children were collected, including blood cells count, inflammatory indicators, organ function, coagulation function, cellular and humoral immunity, pathogenic testing, pulmonary function. Pathogenic tests were performed on each participant after admission by antibodies in blood samples (for MP and Epstein-Barr virus) and nucleic acid in throat swabs (for *Haemophilus influenzae*, *Streptococcus pneumoniae*, Parainfluenza virus, Syncytial virus, etc.). Pulmonary function tests were performed by professional technicians in the asthma center of our hospital before discharge after the acute phase by spirometry, impulse oscillometry (IOS) and tidal breathing flow volume curve (TBFV) analysis, and the results were confirmed by a pulmonologist. All participants underwent lung function testing using the JAEGER Master Screen (Hoechberg, Germany). Predictive values were calculated using the Zepletal predictive value formula for children’s ventilatory function of the machine. According to the recommendations of the guidelines [11–13], TBFV analysis was used for children younger than 3 years old, IOS was used for children aged 3–6 years, and spirometry was used for children older than 6 years old. In spirometry, the normal range for the volume index is greater than 80% of the predicted value and for flow index is greater than 65%. In IOS, the normal range forR_5_ and R_20_ is less than 120% of the predicted value and for Fres is 10 Hz greater than the predicted value. In TBFV, the range of tidal volume (V_T_)/kg is 6–10 ml/kg, and that of time to peak tidal expiratory flow as a proportion of expiratory time (TPTEF/TE, %) and volume to peak tidal expiratory flow as a proportion of exhaled flow (VPEF/VE, %) is 28-55%.

### Statistical analysis

All relevant data were organized in Excel 2016 and statistically analyzed using SPSS version 23.0. Data in different groups were described by means and standard deviations or percentages, depending on the type of variable. Mean and standard deviation (normal distribution of numerical variables), median and interquartile range (abnormal distribution of numerical variables), and percentage (categorical variables) were used for each group of data. The Shapiro‒Wilk test was used to verify the normality of the data. Differences between groups were compared by Student’s t-test or the Mann‒Whitney *U*-test for numerical variables and the chi-squared test for categorical variables. Differences were considered statistically significant when *P* < 0.05.

## Results

### Study population

**Fig. 1:**
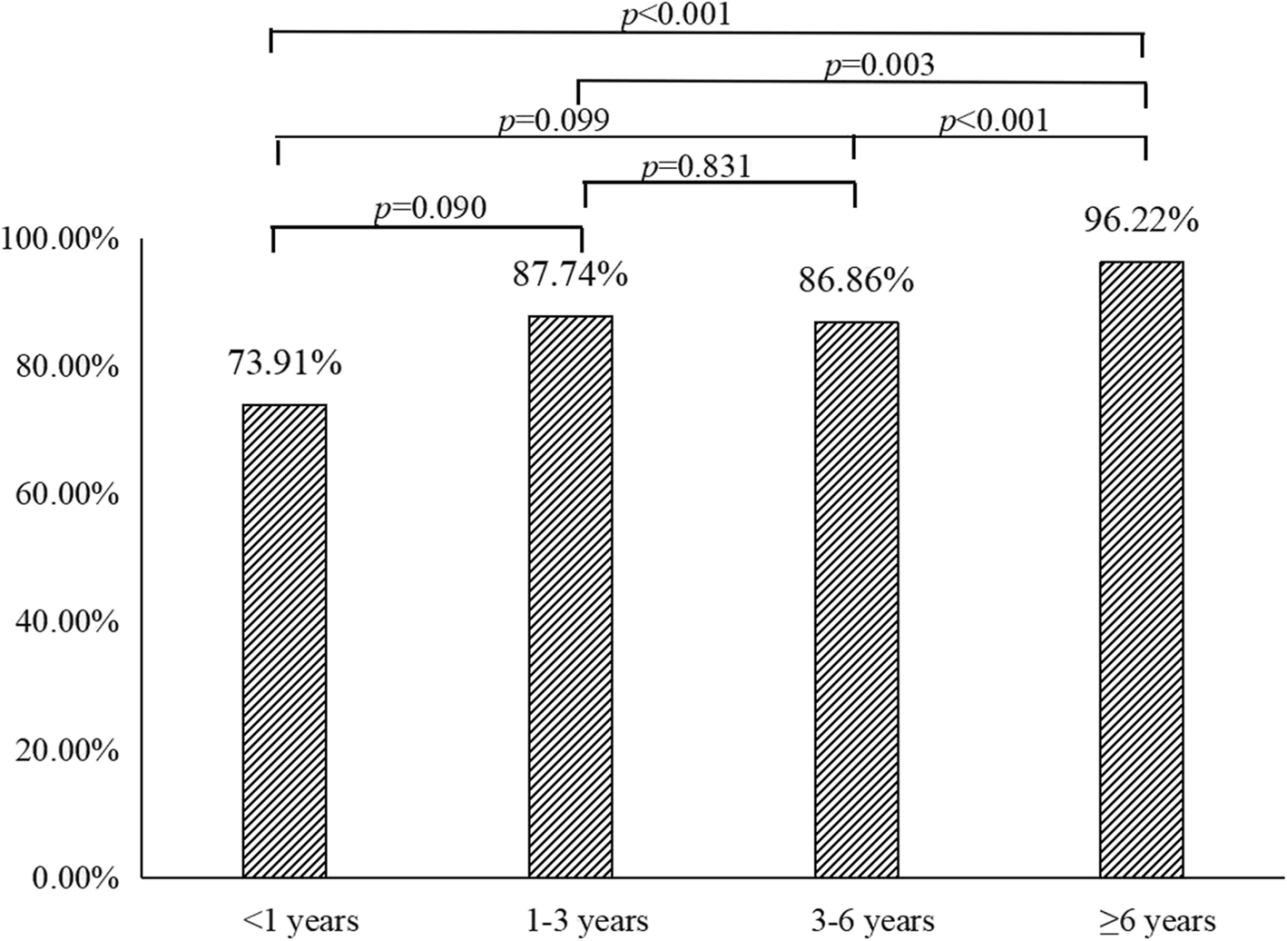
The proportion of fever in different age group (*P* < 0.001)

After excluding unqualified and missing data, a total of 542 children with MPP were included, including 491 children with fever and 51 without fever. The gender distribution showed no statistically significant difference between the two groups (*P* = 0.397). The age of the children with fever ranged from 0.41 to 14 years, with a median age of 5.42 years. The age of the afebrile children ranged from 0.21 to 9 years, with a median age of 3.75 years, and patients in the febrile group were significantly older than those in the afebrile group (*P* < 0.001, Table 1). When the proportions of fever in different age groups were compared, there was the highest proportion of fever in the group older 6 years old (Fig. 1). Table 1 shows the general information, clinical symptoms, imaging information, laboratory results, and pulmonary function.

**Table 1:** General information of MPP children with fever or without fever

	All (*n* = 542)	Febrile Group (*n* = 491)	Afebrile Group (*n* = 51)	*P* value
**General information**				
Male/female	299/243	268/223	31/20	0.397
Age, years	5.21 (3.00, 7.00)	5.42 (3.08, 7.00)	3.75 (1.50, 5.25)	< 0.001
**Clinical symptoms**				
Mixed infection (%)	156 (28.78)	144 (29.3)	12 (23.5)	0.384
Wheeze (%)	116 (21.40)	92 (18.7)	24 (47.1)	< 0.001
**Imaging information**				
Atelectasis (%)	97 (17.89)	93 (18.9)	4 (7.8)	0.049
Pleura effusion (%)	52 (9.59)	50 (10.2)	2 (3.9)	0.232
**Laboratory results**				
WBC count, ×10^9^/L	8.81 (6.72, 11.50)	8.66 (6.43, 11.07)	8.15 (6.49, 12.5)	0.502
PLT, ×10^12^/L	336 (263, 428)	332 (271, 426)	348 (302, 404)	0.045
CRP, mg/L	3.55 (2.86, 9.95)	5.37 (2.86, 11.70)	2.86 (2.86, 3.83)	< 0.001
LDH, U/L	258 (232, 297)	258 (234, 301)	240 (230, 288)	0.335
ESR, mm/h	27 (18, 39)	31 (20, 42)	24 (10, 31)	< 0.001
PCT, ng/ml	0.049 (0.032, 0.086)	0.049 (0.032, 0.087)	0.032 (0.028, 0.048)	0.030
FER, pg/ml	84.1 (64.83, 123)	86.95 (65.20,123.50)	62.75 (31.21, 72.45)	0.040
ALT, U/L	14 (11,19)	14 (11, 19)	12 (9.5, 17)	0.258
CK-MB, ng/dl	1.5 (1,6)	1.38 (1.00, 3.16)	5.535 (1, 10)	0.197
D-dimer, mg/L	0.42 (0.32, 0.65)	0.42 (0.33, 0.65)	0.48 (0.31, 0.77)	0.579
IgE, (g/L)	87.75 (33.23, 281.5)	88.4 (35, 288.5)	67.55 (18.8, 256)	0.357
IgG, (g/L)	9.12 (7.76, 11)	9.24 (7.86, 11,10)	8.19 (6.60, 9.92)	< 0.001
IgA, (g/L)	1.12 (0.73, 1.64)	1.16 (0.765, 1.65)	0.78 (0.36, 1.12)	0.001
IgM, (g/L)	1.39 (1.08 1.85)	1.41 (1.09, 1.85)	1.17 (0.96, 1,71)	0.012
CD4/CD8	1.36 (1.09, 1.75)	1.46 (1.21, 1.87)	1.36 (1.09, 1.75)	0.097

All continuous variables are abnormal distribution and described by median (P25, P75). Categorical variables are described by number (percentage). Data are presented as the mean ± SD (standard deviation) for normal distribution, median (1st quartile, 3rd quartile) for abnormal distribution and n, n (%)

*WBC* white blood cell count, *PLT* platelet, *CRP* C-reactive protein, *LDH* lactate dehydrogenase, *ESR* erythrocyte sedimentation rate, *PCT* procalcitonin, *FER* ferritin, *ALT* alanine aminotransferase, *CK-MB* creatine kinase-MB, *Ig* immunoglobulin

### Clinical characteristics of different groups

In our study, 18.7% (92/491) of patients in the febrile group had wheezing during the course of the disease, which was proportionally lower than the 47.1% (24/51) in the afebrile group. Compared with the afebrile group, the proportion of atelectasis was significantly higher in the febrile group (*P* = 0.049), but there was no significant difference in pleural effusion (*P* = 0.232). Meanwhile, 29.3% (144/491) of children in the febrile group were coinfected with viruses or bacteria, and 23.5% (12/51) of the children in the afebrile group were co-infected with other pathogens. The specific pathogen distribution is shown in Table 2. Epstein-Barr virus (EBV) was more common in febrile group than in afebrile group (*P* = 0.027). There was no significant difference in the rate of other pathogens between the two groups (*P* all > 0.05).

**Table 2:** Pathogen co-infected of the participants

	All (*n* = 542)	Febrile Group (*n* = 491)	Afebrile Group (*n* = 491)	* P* value
**Virus**				
Epstein-barr virus, %	61 (11.25)	60 (12.22)	1 (1.96)	0.027
Parainfluenza virus, %	21 (3.87)	20 (4.07)	1 (1.96)	0.717
Syncytial virus, %	14 (2.58)	14 (2.85)	0 (0.00)	0.448
Influenza B virus, %	12 (2.21)	11 (2.24)	1 (1.96)	1.000
Rhinovirus, %	2 (0.37)	1 (0.20)	1 (1.96)	0.449
Adenovirus, %	2 (0.37)	2 (0.41)	0 (0.00)	1.000
Boca virus, %	1 (0.18)	1 (0.20)	0 (0.00)	1.000
Metapneumovirus, %	1 (0.18)	1 (0.20)	0 (0.00)	1.000
**Bacteria**				
* Haemophilus influenzae*, %	39 (7.20)	34 (6.92)	5 (9.80)	0.636
* Streptococcus pneumoniae*, %	29 (5.35)	24 (4.89)	5 (9.80)	0.247
* Moraxella catarrata*, %	1 (0.18)	1 (0.20)	0 (0.00)	1.000
* Pseudomonas aeruginosa*, %	1 (0.18)	1 (0.20)	0 (0.00)	1.000
* Staphylococcus aureus*, %	1 (0.18)	1 (0.20)	0 (0.00)	1.000

Among all the children with MPP, there was no significant difference in white blood cell count (WBC) between the febrile and afebrile groups (*P* = 0.502). Platelet (PLT), inflammation and humoral immunity indicators such as C-reactive protein (CRP), erythrocyte sedimentation rate (ESR), procalcitonin (PCT), ferritin (FER), immunoglobulin A (IgA), immunoglobulin M (IgM) and immunoglobulin G (IgG), were significantly higher in children with fever than in those without (*P* all <0.05). However, the differences in lactate dehydrogenase (LDH), alanine aminotransferase (ALT), creatine kinase-MB (CK-MB), D-dimer, immunoglobulin E (IgE) and CD4/CD8 between the two groups were not statistically significant (*P* all > 0.05).

There were 84 children who underwent TBFV analysis, 141 by IOS, 154 by spirometry, and 163 children who did not undergo pulmonary function tests for various reasons. Among these, the differences between the two groups were not statistically significant for any of the pulmonary function indicators, neither in value nor in proportion (Table 3, *P* all > 0.05).

**Table 3:** Pulmonary function test of MPP children with fever or without fever

	All	Febrile Group	Afebrile Group	* P* value
**TBFV, n**	84	68	16	
V_T_/kg, mL/kg	9.69 ± 1.87	9.78 ± 1.95	9.20 ± 1.24	0.345
Abnormal, %	2 (2.38)	2 (2.94)	0 (0.00)	1.000
TPTEF/TE, %	20.32 ± 8.88	17.65 ± 6.43	20.95 ± 4.52	0.765
Abnormal	77 (91.67)	62 (91.18)	15 (93.75)	1.000
VPEF/VE, %	23.72 ± 7.39	23.22 ± 5.33	23.06 ± 3.81	0.416
Abnormal, %	67 (79.76)	53 (77.94)	14 (87.50)	0.610
**IOS, n**	141	125	16	
X_5_, kPa/(L·s)	-0.47 ± 0.14	-0.48 ± 0.13	-0.43 ± 0.16	0.202
R_5_, %	107.58 ± 29.67	108.63 ± 28.52	110.55 ± 0.14	0.803
Abnormal, %	33 (23.40)	26 (20.80)	7 (43.75)	0.084
R_20_, %	75.35 ± 16.11	75.34 ± 15.33	76.84 ± 16.77	0.715
Abnormal, %	17 (12.06)	1 (0.80)	0 (0.00)	1.000
R_5_-R_20_, kPa/(L·s)	0.32 ± 0.20	0.33 ± 0.20	0.29 ± 0.19	0.312
Fres (high, %)	73 (51.77)	68 (60.7)	5 (35.7)	0.074
**Spirometry, n**	154	148	6	
FEV_1_, %	88.79 ± 13.59	89.09 ± 13.40	87.93 ± 17.94	0.875
Abnormal, %	41 (26.62)	39 (26.35)	2 (33.33)	1.000
FEV_1_/FVC, %	98.61 ± 6.93	98.84 ± 6.8	94.58 ± 8.94	0.148
Abnormal, %	28 (18.19)	26 (17.57)	2 (33.33)	0.659
PEF, %	79.25 ± 13.99	79.48 ± 14.16	77.91 ± 12.63	0.812
Abnormal, %	16 (10.39)	75 (50.70)	2 (33.33)	0.677
FEF_50_, %	72.05 ± 19.24	72.34 ± 19.15	69.25 ± 26.29	0.717
Abnormal, %	58 (37.66)	56 (37.84)	2 (33.33)	1.000
FEF_75_, %	61.19 ± 22.68	61.43 ± 22.75	55.40 ± 22.19	0.525
Abnormal, %	91 (59.09)	87 (58.78)	4 (66.67)	1.000

All continuous variables are normal distribution and described by median (P25, P75). Categorical variables are described by number (percentage)

*V*_*T*_ tidal volume, *TPTEF/TE (%)* time to peak tidal expiratory flow as a proportion of expiratory time, *VPEF/VE (%)* volume to peak tidal expiratory flow as a proportion of exhaled flow, *FEV*_*1*_ forced expiratory volume in one second, *FEV*_*1*_ */FVC* forced expiratory volume in one second as a proportion of forced vital capacity, *PEF* peak expiratory flow, *FEF*_*50*_ forced expiratory flow at 50 of forced vital capacity, *FEF*_*75*_ forced expiratory flow at 75 of forced vital capacity

## Discussion

In our study, there was an apparent increase in the incidence of MPP from August to December 2021, which is consistent with other studies [14, 15]. The aim of this study was to compare the clinical manifestations of febrile and afebrile children with MP infection. During the 4-month study period, 9.42% (51/542) of the children were afebrile, which is similar to the proportion in a previous study [8]. However, few studies have compared the effect of fever on clinical features, which is unique to this study.

A total of 542 children were included in the study, of whom 491 were febrile and 51 were afebrile. Comparing the general information between the two groups, we found that the patients in the afebrile group were significantly younger than those in the febrile group (*P* < 0.001), suggesting a lower incidence of fever in the younger children with MPP. A study by Sun et al. in 2015 including children under 1 year of age with MP infection showed that 63.89% of older children had fever, while 20% of younger children had fever, with a higher frequency [16]. Other studies including adolescents and other pathogens have also confirmed that the incidence of fever may be lower in infants and younger children [8, 17–20]. This may be because immunity in children improves with age and the immune response is more strongly stimulated in older children [21]. Studies have shown that children over 5 years of age have a relatively more mature immune function than younger children [22], which is consistent with the fact that IgA, IgM and IgG were significantly higher in the febrile group than in the afebrile group in this study (*P* all < 0.05). In addition, we found that IgE levels were increased in both groups, but the difference was not statistically significant (*P* = 0.357). Previous studies have also confirmed that there is indeed an elevated level of IgE in the acute phase of MP infection. In addition to being associated with allergy, MP-infected children with higher IgE levels may have more severe clinical manifestations and complications. IgE may even be a biomarker for complications following MP infection [23]. The immune response is a double-edged sword. On the one hand, an appropriate immune response can activate macrophages in vivo to clear MP from the lung tissue [24]; on the other hand, an excessive immune response can lead to an excessive inflammatory response [22], resulting in severe pneumonia or refractory *Mycoplasma pneumoniae* pneumonia (RMPP).

An interesting finding in our study was that children in the afebrile group had a significantly higher proportion of wheezing than those in the febrile group. However, we do not think that this is due to fever but mainly to age. The febrile group was older, and the afebrile group was younger (5.42 years old to 3.75 years old). It is well known that the mechanism of MP infection can cause airway hyper-responsiveness and increase airway secretions, thereby inducing or exacerbating asthma attacks [25, 26]. Due to the relatively narrow airways, infants are more likely to have airway hypersecretion and hyper-responsiveness after respiratory infection and are more likely to wheeze [27–29]. Therefore, we believe that the wheeze rate of the afebrile group is significantly higher than that of the febrile group, which is related to the physiological and pathological characteristics of age. There was no significant difference between the two groups in the proportion of mixed infections overall and for each pathogen (except EBV). The rate of fever in children infected with EBV is relatively high. However, there are no reports about that EBV infection is associated with wheezing and impairment of pulmonary function, so co-infection isn’t considered an influencing factor.

In this study, inflammatory indicators, including CRP, PCT, ESR, and FER, were significantly higher in the febrile group than in the afebrile group, indicating that the inflammatory response was significantly stronger in children with fever and older age, which is basically consistent with previous literature [16, 19, 30]. There was no significant difference in the increase in LDH between the two groups. LDH is an enzyme involved in glycolysis. LDH is released from cells into the blood during the inflammatory response, and its level may reflect the intensity of the inflammatory response and the severity of organ damage [31]. Several studies have found that elevated LDH is a major risk factor for RMPP and postinfectious obliterans [31–34], suggesting that children who are not febrile are equally likely to develop severe disease and have a poor prognosis. In addition, there was no significant difference in ALT, CK-MB or D-dimer, indicating that fever was not associated with organ dysfunction or coagulation abnormalities.

However, beyond expectations, there were no apparent significant differences between the two groups in all the pulmonary function parameters. Eighty-four children underwent TBFV analysis. IOS was performed in all 141 children, and spirometry was performed in 154. In the TBFV analysis used in the younger age group, VT was normal, while TPTEF/TE and VPEF/VE decreased significantly, suggesting that the children in the younger age group had moderate obstructive ventilatory dysfunction. X_5_ in IOS was significantly lower than normal, indicating peripheral small airway dysfunction in all children, while R_5_ and R_20_ were in the normal range, indicating that the total airway pressure, especially the central airway pressure, was not significantly affected. There was no significant difference in the difference in R_5_ and R_20_ between the two groups. In addition, in spirometry, FEV_1_ and FEV_1_/FVC were all within the normal range, but FEF_75_ was decreased in both groups, suggesting that the children in this group also had small airway dysfunction. MP infection can cause obstructive airway dysfunction [35]. A large number of studies have confirmed that the reduction is mainly in small airways [36], and our research data are also consistent with this finding. It is not difficult to see that there was no significant difference in pulmonary function between the two groups. Regardless of the type of pulmonary function test, small airway function decreased significantly in both the febrile and afebrile groups. This means that patients without fever also need to be taken into account, even more than those without fever.

This is a single-center retrospective study with a small sample size, which may have some bias. In addition, the types of pulmonary functions performed were different due to the age of the children, and the number of each pulmonary function test performed was relatively small, which may affect the statistical results. Data on treatment and follow-up are not included in this study, which is our future development direction. As this is a retrospective observational study, there are some confounding factors such as age, co-infection although they have been discussed. A prospective study is needed to confirm this.

This study retrospectively analyzed demographic data, the presence of wheezing or atelectasis, laboratory results and pulmonary function. An important conclusion is drawn: MP infection is more common in older children and fever is more prominent. The rate of fever is lower in younger children, but the rate of wheezing is higher than that in older children. In afebrile children, although the inflammatory indicators were not as high, the degree of the impairment of organ and lung function was no less than in febrile children. Therefore, attention should also be paid to children who are not febrile.

## Data Availability

All data generated or analyzed during this study are included in this published article.

## Abbreviations

MP: *Mycoplasma Pneumoniae*
MPP: *Mycoplasma pneumoniae* pneumonia
CAP: Community-acquired pneumonia
EBV: Epstein-Barr virus
IOS: Impulse oscillometry
TBFV: Tidal breathing flow volume curve
WBC: White blood cell count
PLT: Platelet
CRP: C-reactive protein
ESR: Erythrocyte sedimentation rate
PCT: Procalcitonin
FER: Ferritin
Ig: Immunoglobulin
LDH: Lactate dehydrogenase
ALT: Alanine aminotransferase
CK-MB: Creatine kinase-MB
VT: Tidal volume
TPTEF/TE (%): Time to peak tidal expiratory flow as a proportion of expiratory time
VPEF/VE (%): Volume to peak tidal expiratory flow as a proportion of exhaled flow
FEV_1_: Forced expiratory volume in one second
FEV_1_/FVC: Forced expiratory volume in one second as a proportion of forced vital capacity
PEF: Peak expiratory flow
FEF_50_: Forced expiratory flow at 50 of forced vital capacity
FEF_75_: Forced expiratory flow at 75 of forced vital capacity
